# Unbalanced segregation of a paternal t(9;11)(p24.3;p15.4) translocation causing familial Beckwith-Wiedemann syndrome: a case report

**DOI:** 10.1186/s12920-019-0539-y

**Published:** 2019-06-07

**Authors:** Caroline Lekszas, Indrajit Nanda, Barbara Vona, Julia Böck, Farah Ashrafzadeh, Nahid Donyadideh, Farnoosh Ebrahimzadeh, Najmeh Ahangari, Reza Maroofian, Ehsan Ghayoor Karimiani, Thomas Haaf

**Affiliations:** 1 Institute of Human Genetics, Julius Maximilians University Würzburg, Biozentrum, Am Hubland, 97074 Würzburg, Germany; 2 Department of Pediatric Diseases, Mashhad University of Medical Sciences, Mashhad, Iran; 3 Department of Internal Medicine, Mashhad University of Medical Sciences, Mashhad, Iran; 4 Department of Modern Sciences and Technologies, Mashhad University of Medical Sciences, Mashhad, Iran; 5 Molecular and Clinical Sciences Institute, St. George’s University of London, Cranmer Terrace, London, UK

**Keywords:** Familial Beckwith-Wiedemann syndrome, Copy number variation, Duplication-deficiency, Genomic imprinting, Submicroscopic chromosome rearrangement, Reciprocal translocation

## Abstract

**Background:**

The vast majority of cases with Beckwith-Wiedemann syndrome (BWS) are caused by a molecular defect in the imprinted chromosome region 11p15.5. The underlying mechanisms include epimutations, uniparental disomy, copy number variations, and structural rearrangements. In addition, maternal loss-of-function mutations in *CDKN1C* are found. Despite growing knowledge on BWS pathogenesis, up to 20% of patients with BWS phenotype remain without molecular diagnosis.

**Case presentation:**

Herein, we report an Iranian family with two females affected with BWS in different generations. Bisulfite pyrosequencing revealed hypermethylation of the *H19*/*IGF2*: intergenic differentially methylated region (IG DMR), also known as imprinting center 1 (IC1) and hypomethylation of the *KCNQ1OT1*: transcriptional start site (TSS) DMR (IC2). Array CGH demonstrated an 8 Mb duplication on chromosome 11p15.5p15.4 (205,827-8,150,933) and a 1 Mb deletion on chromosome 9p24.3 (209,020-1,288,114). Chromosome painting revealed that this duplication-deficiency in both patients is due to unbalanced segregation of a paternal reciprocal t(9;11)(p24.3;p15.4) translocation.

**Conclusions:**

This is the first report of a paternally inherited unbalanced translocation between the chromosome 9 and 11 short arms underlying familial BWS. Copy number variations involving the 11p15.5 region are detected by the consensus diagnostic algorithm. However, in complex cases which do not only affect the BWS region itself, characterization of submicroscopic chromosome rearrangements can assist to estimate the recurrence risk and possible phenotypic outcomes.

## Background

Beckwith-Wiedemann syndrome (BWS; MIM #130650) is a clinically variable overgrowth syndrome with a prevalence of 1:10,340 live-births [1, 2]. Cardinal features of the consensus Beckwith-Wiedemann spectrum (BWSp) scoring system [3] include macroglossia, exomphalos, lateralized overgrowth, hyperinsulinism, and predisposition to embryonal tumors (e.g. multifocal and/or bilateral Wilms tumor or nephroblastomatosis). The BWS locus on chromosome 11p15.5 contains several genes involved in cell proliferation, which are regulated by two differentially methylated regions (DMRs), which function as imprinting centers (ICs). The paternally methylated *H19*/*IGF2*:IG DMR (IC1) controls paternal expression of the insulin-like growth factor 2 (*IGF2*), which is implicated in growth and tumorigenesis, and maternal expression of *H19*, a non-coding RNA, which restricts growth by way of a *cis* control on *IGF2* and may also have a tumor suppressor function. The maternally methylated *KCNQ1OT1*:TSS DMR (IC2) controls paternal expression of the long non-coding RNA *KCNQ1OT1*, which in turn silences *KCNQ1* and presumably also *CDKN1C* in *cis* via chromatin remodeling. The cyclin-dependent kinase inhibitor 1C (*CDKN1C*) is a negative regulator of cell proliferation [4–9].

There is a complex interplay of paternally expressed growth-promoting (*IGF2* and *KCNQ1OT1*) and maternally expressed growth-inhibiting factors (*H19* and *CDKN1C*) in the BWS region. BWS can arise through various molecular mechanisms. Aberrant hypomethylation of IC2 accounts for approximately 50%, segmental paternal uniparental disomy (UPD) for ~ 20%, loss-of-function mutations of the maternal *CDKN1C* gene for 5–10%, and hypermethylation of IC1 for ~ 5% of patients. The majority (80–85%) of BWS cases occur sporadically [3, 8, 9]. Familial forms (15–20%) may be caused by maternal loss-of-function *CDKN1C* mutations, balanced chromosomal rearrangements involving the maternal chromosome 11p15.5, maternal deletions and OCT4/SOX2 binding site mutations within IC1, or copy number variations (CNVs) of the paternal allele [8–11]. Because of its broad phenotypic spectrum and overlap with other overgrowth syndromes, the clinical diagnosis of BWS is challenging. Best practice guidelines have been developed for a standardized clinical and molecular diagnostics and management of patients with Beckwith-Wiedemann spectrum [3]. Here, we describe an Iranian family with BWS-affected individuals in two generations due to a familial reciprocal translocation t(9;11)(p24.3;p15.4).

## Case report

**Fig. 1:**
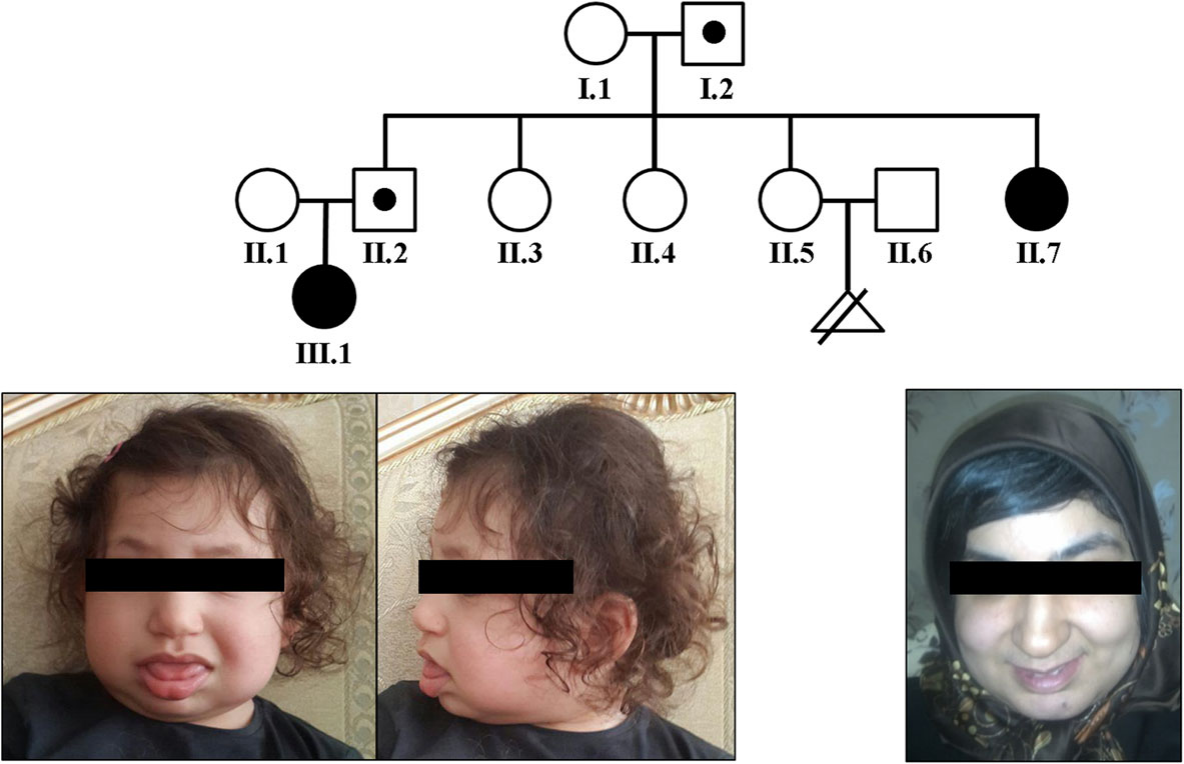
The upper part shows the pedigree of a three-generation Iranian family with two females affected with BWS (black circles). Balanced translocation carriers are represented by a dot in the middle of the symbol. The photographs below show the two-year old girl III.1 and the 36-year-old woman II.7, exhibiting macroglossia and typical facial appearance of BWS

An Iranian family presented with two females (aunt and niece) affected with BWS in different generations (Fig. 1). The aunt II.7 was born to a healthy couple as the last of five children. The pregnancy was unremarkable, except for accelerated intrauterine growth. Sporadic seizures during childhood were medicated and since then she has been seizure-free. Because of severe anemia she was treated with folic acid. Now at age 36, she exhibits a round face with full cheeks, macroglossia, and intellectual disability (ID). The niece III.1 is the first child of the oldest brother of female II.7 with BWS. She (III.1) was delivered at 35 weeks of gestation with a birth length of 47 cm (Z score 1) and a birth weight of 3150 g (Z score 2). Apart from oligohydramnion, no pregnancy-related medical problems were observed. At birth she showed microcephaly, a round face with full cheeks, a broad nasal bridge, and macroglossia. Starting from the age of 15 months, she exhibited recurring seizures and upper respiratory tract infections. Now at an age of two years, she displays global developmental delay and agitation.

### Bisulfite pyrosequencing

Genomic DNA was extracted from whole blood following the standard salt extraction method and bisulfite converted with the EpiTect Fast Bisulfite Conversion Kit (Qiagen, Hilden, Germany). PCR and sequencing primers (Table 1) were adopted from Paganini et al., 2015 [12]. Amplification was performed with an initial denaturation step at 95 °C for 5 min, 40 cycles of 95 °C for 30 s, primer-specific annealing temperature (54 °C for IC1 and 57 °C for IC2) for 30 s, 72 °C for 45 s, and a final extension step at 72 °C for 5 min. Bisulfite pyrosequencing was performed on a PyroMark Q96 MD Pyrosequencing System using the PyroMark Gold Q96 CDT Reagent Kit (Qiagen) and 0.5 μl of sequencing primers (10 μM). Data analysis was done with the Pyro Q-CpG software (Qiagen).

**Table 1:** PCR and sequencing primers^a^ for bisulfite pyrosequencing

Region	Primer	Sequence (5′ to 3′)	CpGs	Chromosomal localization (bp)^b^	Amplicon size (bp)	Sequence to analyze
*ICR1*	Forward	TGGGTATTTTTGGAGGTTTTTTT	4	Chr11: 2,020,978 - 2,021,291	314	YGYGTYGTAGGGTTTAYGGGGGTT
	Reverse	^c^AACTTAAATCCCAAACCATAACA				
	Sequencing	GTTTYGGGTTATTTAAGTTA				
*ICR2*	Forward	TTGTTTATAAGGTGTAGATGGGAG	4	Chr11: 2,720,465 - 2,720,669	205	AYGTTTGTGATTTGGGAYGGTYGYGGGGTATATAGTTTATT
	Reverse	^c^TCTCCCAAACTCTCCTCAAC				
	Sequencing	TAGGTTAGGTTGTATTGTTG				

^a^Adopted from Paganini et al., 2015 [12]

^b^Genomic coordinates are based on Ensembl release 75

^c^Primer is biotinylated at the 5′ end

**Fig. 2:**
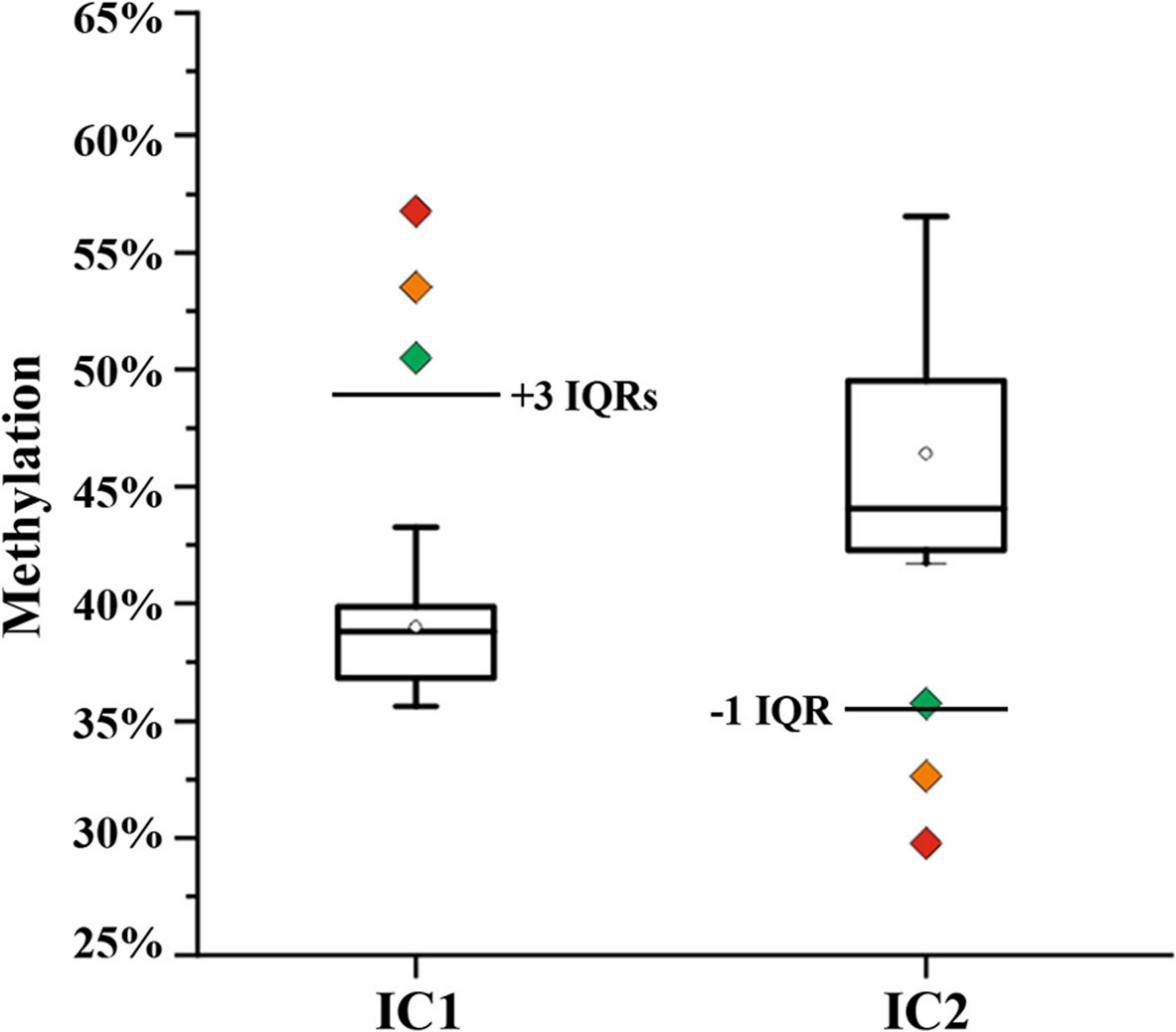
Mean methylation values of IC1 and IC2, measured by bisulfite pyrosequencing, in one BWS sample with mosaic upd(11p15.5)pat, indicated by a green diamond, and the two affected family members II.7 and III.1, indicated by orange and red symbols. The black box plots demonstrate methylation variation among 7 control samples without BWS. The bottom of the box indicates the 25^th^ percentile, the top the 75^th^ percentile. The median is represented by a horizontal line and the mean by an open diamond symbol within the box. Whiskers represent the observed methylation range in normal samples

The mean methylation β values (duplicate measurements of 4 CpGs each for IC1 and IC2) were compared between the two study samples, one BWS sample with upd(11p15.5)pat, and 7 normal controls. We found hypermethylation of IC1 and hypomethylation of IC2 in both affected individuals II.7 (IC1 56.8%, IC2 29.8%) and III.1 (IC1 53.5%, IC2 32.6%), compared to normal controls (mean IC1 38.5%, IC2 45.4%). The gain of methylation by ≥ 15 percentage points (compared to the mean of controls) at IC1 is consistent with the presence of two methylated paternal and one unmethylated maternal allele(s), whereas the loss of methylation by > 12 percentage points at IC2 is due to two unmethylated paternal and one methylated maternal allele(s). Methylation (IC1 50.5%, IC2 35.8%) of the upd(11p15.5)pat sample is consistent with a mosaic UPD with a large proportion of normal cells. For a non-mosaic upd(11p15.5)pat spanning both domains one would expect IC1 methylation levels > 80% and IC2 methylation levels < 20% by bisulfite pyrosequencing. The box plots in Fig. 2 demonstrate the range of methylation variation in normal individuals. At IC1 (median 38.8%, IQR 3.0%) the methylation measurements of the three BWS patients are more than three interquartile ranges (IQRs) away from the box, whereas at IC2 (median 44.0%, IQR 7.2%) the distance is between one and two IQRs. However, the larger normal variation at IC2 is mainly due to samples in the third and fourth quartiles. The analyzed BWS samples are clearly hypomethylated at IC2. Between-group comparisons revealed a significant difference (T test; *p* < 0.0001 for IC1 and *p* = 0.003 for IC2) between BWS samples and healthy controls.

### Molecular cytogenetic analyses

**Fig. 3:**
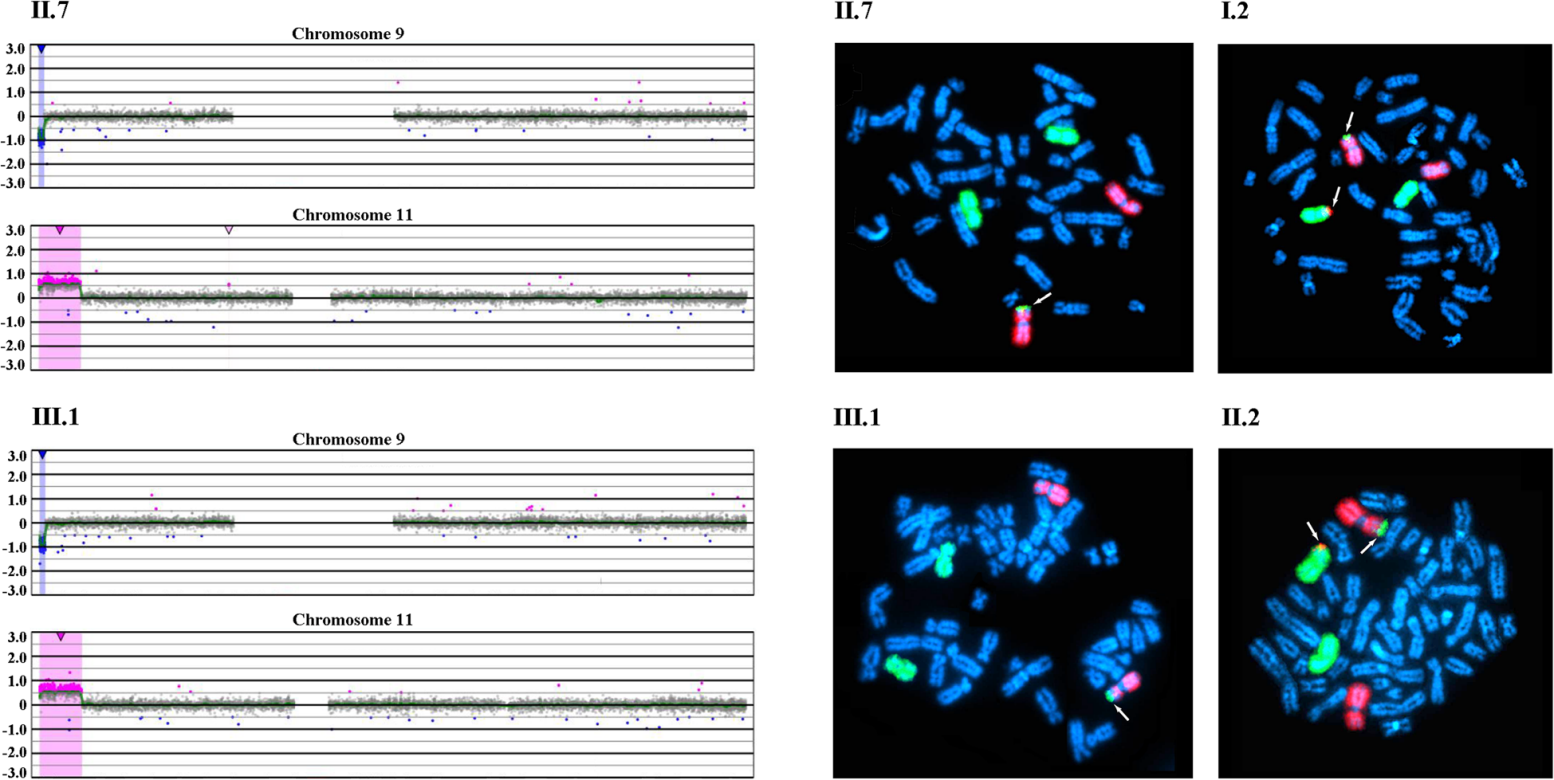
The left side shows the results of array CGH analysis of females II.7 and III.1, affected with BWS. The hybridization profiles are consistent with a heterozygous loss of chromosome 9p24.3, (209,020-1,288,114) × 1, and a heterozygous gain of chromosome 11p15.5p15.4, (205,827-8,150,933) × 3. The right side shows representative metaphase spreads of the affected females and their fathers, hybridized with chromosome 9 (red) and chromosome 11 (green) painting probes. II.7 and III.1 display a derivative chromosome 9 with chromosome 11 material on the short arm. The fathers I.2 and II.2 are endowed with a reciprocal translocation between chromosomes 9p and 11p

Array comparative genomic hybridization (CGH) was performed using the CGX DNA labeling kit (PerkinElmer, Rodgau, Germany) and the CGX-HD array chip (PerkinElmer) that covers clinically relevant regions with 180,000 oligonucleotide markers. A female genomic DNA sample served as a reference. The hybridized chip was scanned with the NimbleGen MS-200 Microarray Scanner (Roche Diagnostics, Mannheim, Germany). Data analysis was conducted with CytoGenomics 2.5 (Agilent, Waldbronn, Germany) and Genoglyphix 3.0 (PerkinElmer) software using annotations from human genome assembly GRCh37. Chromosomal microarray analysis revealed a 7.95 Mb heterozygous copy number gain including the entire BWS-critical region on chromosome 11p15.5p15.4 (arr[hg19] 11p15.5p15.4(205,827-8,150,933) × 3), along with a 1.08 Mb heterozygous copy number loss of chromosome 9p24.3 (arr[hg19] 9p24.3(209,020-1,288,114) × 1) in affected individuals II.7 and III.1 (Fig. 3, left side).

Chromosomes were prepared from lymphocyte cell cultures according to standard protocols. Fluorescence in situ hybridization (FISH) was performed with XCyting Chromosome Paints (MetaSystems, Altlussheim, Germany) for chromosomes 9 and 11. Hybridized chromosome slides were analysed using an epifluorescence microscope Axio imager A1 (Carl Zeiss, Jena, Germany), equipped with a FISHView system (Applied Spectral Imaging, Edingen-Neckarhausen, Germany). At least ten metaphases of each proband were evaluated. FISH analysis with chromosome 9 (red) and 11 (green) painting probes revealed a derivative chromosome 9 with chromosome 11 material on the short-arm subtelomere in both affected females (II.7 and III.1) (Fig. 3, right side). The father (I.2) of the aunt (II.7) and the father (II.2) of the niece (III.1) showed a reciprocal exchange of material between chromosomes 9 and 11, consistent with a reciprocal t(9;11)(p24.3;p15.4) translocation (Fig. 3, right). The mothers (I.1 and II.1) of both affected females showed normal FISH karyotypes.

## Discussion and conclusion

The main cause (~ 40%) of familial BWS are maternal loss-of-function mutations in *CDKN1C*. The remaining cases are due to maternally inherited balanced translocations and inversions with breakpoints in chromosome 11p15.5 or to paternally derived 11p15.5 duplications, which may be the unbalanced segregation products of familial balanced translocations [3, 8, 9]. It has been hypothesized [13] that maternal balanced rearrangements interfere with epigenetic resetting of the BWS-critical region in the female germline by a *cis*-acting element(s). This view is supported by several publications, describing individuals with BWS and balanced maternal t(11;17)(p15.5;q21.3) and t(9;11)(p11.2;p15.5) translocations [14, 15]. A number of paternal reciprocal translocations associated with 11p15.5 duplications in the affected children have been reported [13, 16–18]. Recurring translocations, in particular between 5p and 11p may be driven by sequence homologies. In a large study using methylation-specific multiplex ligation-dependent probe amplification [8], 14 (8%) of 167 patients with the molecular diagnosis of BWS exhibited CNVs in the 11p15.5 region. One of 6 duplication patients exhibited a paternally inherited unbalanced t(4;11) translocation.

Consistent with best practice guidelines [3, 9], the BWS diagnosis in the two affected females of our family was first confirmed by methylation testing. Bisulfite pyrosequencing is a relatively simple but highly accurate method for quantitative methylation measurements. In our experience, the methylation difference between technical replicates (including bisulfite conversion) is in the order of 1–2 percentage points. Since pyrosequencing provides the mean methylation of a large number of DNA molecules in a genomic DNA sample, it does not distinguish between paternal and maternal allele methylation of imprinted alleles. Theoretically, one would expect 50% methylation of imprinted genes with one methylated and one unmethylated allele. However, the measured methylation values do not only depend on the methylation status of a given CpG(s) but also on assay design (that can lead to biased amplification of either the unmethylated or the methylated allele), genetic variation, and parental factors [19]. Nevertheless, the measured methylation levels indicated clear hypermethylation of IC1 and hypomethylation of IC2 in both affected females and a mosaic upd(11p15.5)pat, compared to normal controls. The described assay is inexpensive, fast and can be easily adopted in molecular genetic laboratories, however before application in routine diagnostics it needs to be validated on a larger number of BWS and control samples. Based on our preliminary analysis of 3 BWS and 7 control samples we suggest that hypermethylation or hypomethylation by ≥ 10 percentage points is indicative of abnormal methylation patterns. Methylation variation at IC1 among normal individuals appears to be smaller than that at IC2 and, consequently, the sensitivity of the assay may be better for IC1. It is interesting to note that methylation at both IC1 and IC2 differs by approximately 3 percentage points between the niece and aunt carrying the same unbalanced translocation. This is likely due to confounding factors such as age and BMI [19–21].

The combination of 11p15.5p15.4 duplication and 9p24.3 deletion, detected by array CGH in our patients was consistent with an unbalanced meiotic segregation of a balanced paternal translocation. Indeed a reciprocal t(9p;11p) translocation was demonstrated by chromosome painting in the unaffected fathers I.2 and II.2. Male balanced translocation carriers (I.2 and II.2) have a recurrence risk up to 25% for a child with BWS due to a 11p15.5p15.4 duplication and 9p24.3 deficiency. The reciprocal condition of a 11p15.5p15.4 deletion involving 131 OMIM genes and 9p24.3 duplication may cause pregnancy loss (as observed in the possible translocation carrier II.5) or a child with syndromal ID and a phenotype more severe than BWS (see https://decipher.sanger.ac.uk). Chromosome analysis of the healthy sisters (II.3. II.4, and II.5) of the affected aunt (II.7) was recommended.

It has been reported [6, 17, 18, 22] that patients with BWS due to a paternally inherited 11p15.5 duplication exhibit macroglossia, distinct craniofacial features, including prominent occiput and forehead, a round face with full cheeks, broad and flat nasal bridge, micrognathia, hypertelorism, deep set eyes with epicanthus as well as an increased risk for ID. Although our two patients fit well to this description, the considerable size variation of the duplicated 11p15.5 segments and additional chromosomal imbalances in some patients render such genotype-phenotype correlations difficult. We cannot exclude that part of the symptoms in our patients are caused or modulated by heterozygous loss of 9p material, including the OMIM genes *DOCK8*, *KANK1*, *DMRT1*, *DMRT2*, and *DMRT3*, or the gain of 11p material, involving 131 OMIM genes, including the entire beta globin locus. A duplication of the Hbb (epsilon-, gamma-G-, gamma-A-, delta-, and beta-globin) gene cluster has been reported in a women with sickle cell-like anemia [23] and could also contribute to the severe anemia in the aunt with BWS. Homozygous or compound heterozygous mutations in the dedicator of cytokinesis 8 (*DOCK8*) gene cause hyper-IgE syndrome [24]. It is possible that *DOCK1* haploinsufficiency contributes to recurrent bacterial infections in one of our patients. Paternal deletions of the KN motif and ankyrin repeat domains 1 (*KANK1*) gene have been associated with cerebral palsy spastic quadriplegic type 2 (CPSQ2), a severe central nervous development disorder [25]. The imprinted-like behaviour of paternal *KANK1* deletions was explained by hypomethylation of the neighboring *DMRT1* gene, leading to expression of an antisense transcript, which represses *KANK1* in *trans*. Since the *DMRT* genes in *cis* are deleted in our patients, they do not present with CPSQ2. Both gains and losses of *KANK1* have been associated with childhood seizures and developmental delay [26] which are seen in our two patients but are not typical for BWS. Haploinsufficiency of doublesex- and mab3-related transcription factors (*DMRT*) in 9p24.3 has been associated with failure of testicular development and XY sex reversal [27, 28]. Females display a wide phenotypic spectrum ranging from primary ovarian failure to mild gonadotropin hyperresponsiveness, normal genitalia and pubertal development [29, 30]. Our patients showed normal sexual development by age.

The classical monosomy 9p syndrome (MIM #158170), which is characterized by trigonocephaly, midface hypoplasia, long philtrum, hypotonia and ID results from chromosome 9p22p23 deletions [31]. Thus, the critical region lies proximal of the 9p24.3 segment which is deleted in our patients. Overall, reciprocal translocations between the chromosome 9 and 11 short arms appear to be very rare. A balanced t(9;11)(p11.2;p15.5) translocation in a girl with BWS was inherited from her phenotypically normal mother, who was a de novo translocation carrier [15]. A balanced t(9;11)(p21.2;p14.2) translocation not affecting the BWS-critical region was reported in a neonate with epicanthal folds, flat nasal bridge, small mouth, micrognathia, low-set ears, and cleft palate [32]. Her phenotype, which does not include features of BWSp, may be caused by a disrupted gene(s) and/or microdeletions in the breakpoint regions.

In the BWS family described here the results of methylation analysis were consistent with a paternal 11p15.5 duplication. Array CGH revealed that BWS in the two affect females was due to an unbalanced segregation of a paternal reciprocal t(9;11)(p24.3;p15.4) translocation. BWS can be caused by both balanced and unbalanced 11p15.5 translocations originating from either parent. Unbalanced translocations will be detected by assessing CNVs in the 11p15.5 region, which is part of the recommended diagnostic algorithm for BWS [3]. Conventional chromosome analyses are usually performed in familial cases of BWS, especially those without *CDKN1C* mutations. Since the recurrence risk of BWS and possible phenotypic outcomes depend on size, gene content, copy number, and parental inheritance of the involved chromosome regions, it is reasonable to characterize the underlying submicroscopic chromosome rearrangements in translocation carriers and their families.

## Data Availability

All relevant data are contained within the manuscript.

## Abbreviations

BWS: Beckwith-Wiedemann syndrome
BWSp: Beckwith-Wiedemann spectrum
CGH: Comparative genomic hybridization
CNV: Copy number variation
FISH: Fluorescence in situ hybridization
IC: Imprinting center
UPD: Uniparental disomy
